# Persistence of *L. V. braziliensis* in the Nasal Mucosa of Treated Patients

**DOI:** 10.3390/biomedicines13071634

**Published:** 2025-07-03

**Authors:** Jackeline Maria de Sousa Lima Lopes, Aline de Fatima Filha Santos, Renata Gabriella Ribeiro Ferreira, Thalion Gabriel Alves Moreira, Veronica Maria Gonçalves Furtado, Keven Styvenn Brito Santana, Thallyta Maria Vieira, Daniel Holanda Barroso, Sílvio Fernando Guimarães de Carvalho, Raimunda Nonata Ribeiro Sampaio

**Affiliations:** 1 Reference Center for Infectious Diseases, University of Montes Claros, Montes Claros 39400-351, MG, Brazil; jackelinellima@hotmail.com (J.M.d.S.L.L.); thaliongam@gmail.com (T.G.A.M.); thallyta.vieira@unimontes.br (T.M.V.); silvio.carvalho@unimontes.br (S.F.G.d.C.); 2 Postgraduate Program in Medical Sciences, School of Medicine, University of Brasília, Brasília 70910-900, DF, Brazil; alinefilha2012@gmail.com (A.d.F.F.S.); veronicafurtado.orquidea@gmail.com (V.M.G.F.); kevennsty@gmail.com (K.S.B.S.); rnrsampaio@hotmail.com (R.N.R.S.); 3 Faculty of Medicine, University of Brasília, Brasília 70910-900, DF, Brazil; 4 University Hospital of Brasília, University of Brasília, Brasília 70910-900, DF, Brazil; 5 Laboratory of Dermatomycology, Faculty of Medicine, University of Brasília, Brasília 70910-900, DF, Brazil; 6 Postgraduate Program in Health Sciences, School of Health Sciences, University of Brasília, Brasília 70910-900, DF, Brazil

**Keywords:** leishmaniasis, prognosis, real time polymerase chain reaction, cutaneous leishmaniasis

## Abstract

**Background/Objectives**: Cutaneous leishmaniasis is an infectious disease that most frequently affects neglected populations. Besides its incidence, a high disease burden is associated with the possibility of mucosal sequelae. Clinical follow-up of these patients is difficult due to the limited access of the affected population to healthcare and the long lapse between the development of cutaneous and mucosal diseases. In this study, we evaluated the positivity of *L. V. braziliensis* DNA on the nasal mucosa of patients treated for leishmaniasis in an attempt to estimate the possible long-term risk of developing mucosal leishmaniasis and its association with important clinical characteristics. **Methods**: Samples were collected immediately after treatment completion using a nasal swab and specific DNA was amplified and detected using real-time PCR. Clinical and laboratorial data was systematically collected. **Results**: The positivity of *L. V. braziliensis* was 7% after treatment, and of this 60% had mucosal lesions before treatment, compared with only 13.4% in patients negative for *L. V. braziliensis* after treatment (*p* = 0.031). **Conclusions**: Molecular detection of *L. V. braziliensis* DNA on the nasal mucosa is a promising strategy to improve the follow-up and treatment of patients with American Tegumentary Leishmaniasis.

## 1. Introduction

The introduction Leishmaniasis is a significant world health problem with an estimated global yearly incidence of 0.6 to 1 million cases [1,2]. Beyond its preponderance in vulnerable populations [2], this disease can evolve in a chronic way and lead to sequalae [3,4,5]. Mucosal lesions can occur in isolation or can be associated with contemporary cutaneous lesions [6]. Roughly 90% of mucous leishmaniasis (ML) cases are from Bolivia, Brazil, Ethiopia and Peru [2], and a high number of cutaneous lesions, age, sex and disease duration are the main risk factors for this form [7,8].

ML most frequently compromises the nasal mucosa, specially the nasal septum [9], but it can damage the lips, mouth, pharynx, larynx and even the bronchia [10,11,12,13,14]. Common clinical complaints are epistaxis, persistent rhinorrhea, nasal obstruction [15], sores in the oral cavity, odynophagia, dysphagia, dysphonia and cough [16,17]. The lesion commonly starts with an erythema and infiltration in the nasal mucosa followed by the formation of a granulation tissue and posterior ulceration [18], from which can follow nasal septal perforation and a compromise of the supporting structures of the nose, finally resulting in saddle nose or *facies leishmaniotica* [6]. Worrisomely, the majority of patients have ulcerated and destructive lesions when diagnosed [19].

Among the challenges in the prevention of secondary sequelae resulting from mucosal compromise are the time gap between the emergence of a cutaneous lesion and the diagnosis of mucosal compromise [15], which is a result of its oligosymptomatic initial characteristic and the fact that the initial lesion is on an area not immediately seen by the patient. One of the possible ways to screen patients with a higher risk of developing mucosal disease in the future is to search for the parasite in the nasal mucosa using real-time PCR [20]. In this study, we aim to evaluate the presence of *L. V. braziliensis* DNA in the nasal mucosa of patients attending tertiary hospitals in Brazil, associating this positivity with relevant clinical outcomes.

## 2. Materials and Methods

We conducted a longitudinal study with patients who attended the Leishmaniasis Clinic at the University Hospital of Brasilia and Montes Claros State’s University—UNIMONTES—which are referral centers for leishmaniasis diagnosis and treatment in the Brazilian mid-western and southeastern regions, between March 2023 and March 2024. Initially, patients with active lesions suggestive of leishmaniasis were screened for inclusion, with the collection of clinical and laboratorial data. After the initial investigation, patients under immunosuppressive therapy or without a confirmed diagnosis of American Tegumentary Leishmaniasis (ATL) were excluded, and only patients with lesions confirmed to be ATL were included. All patients were tested for HIV, with none of them being positive, and a complete blood count with differential was conducted, with none presenting with Common Terminology Criteria for Adverse Events hematologic thresholds: hemoglobin less than 10.0 g per deciliter, white blood cells less than 3.0 × 10^9^ per liter, neutrophils less than 1.5 × 10^9^ per liter, lymphocytes less than 0.8 × 10^9^ per liter, and platelets less than 75,000 per microliter. Confirmation of leishmaniasis diagnosis was based on the results of clinical evaluation, indirect immunofluorescence, direct skin exam, culture of skin aspirates in Novy–MacNeal–Nicolle medium and polymerase chain reactions of skin fragments as described by Gomes at al [21]. After inclusion criteria were met, patients were submitted to a standardized clinical evaluation which included collecting patient characteristics (age and sex), epidemiological information (occupation and whether they resided in a rural region) and disease characteristics (number of lesions and presence of nasal symptoms). All patients were also examined by the assistant dermatologist with anterior rhinoscopy and oroscopy to access possible mucosal lesions. Anterior nasal swabs were also collected before the initiation and immediately after the complete course of treatment. On the 90th day after the beginning of treatment, patients were re-evaluated for clinical cure, this being considered complete re-epithelialization of lesions associated with complete flattening. Treatment allocation was performed according to clinical criteria established by the assistant team following the Brazilian ministry of health guidelines [22]. Individuals responsible for the performance of molecular tests were blinded to patients’ clinical characteristics.

Anterior nasal swabs (CATCH-ALL™ sample collection swab, Promega, Madison, WI, USA) were collected from all included patients. The swab was rotated five times in each nasal fossa at the anterior septum and inferior turbinate head. DNA extraction from the nasal swab samples was performed using the PureLink Genomic DNA Kit (Invitrogen, Carlsbad, CA, USA) according to the manufacturer’s protocol. Real-time quantitative polymerase chain reactions (qPCR) were performed with TaqMan specific primers for *L. V. braziliensis* detection (forward 5′-TGCTATAAAATCGTACCACCCGACA-3′, reverse 5′-GAACGGGGTTTCTGTATGCCATTT-3′), and probe FAM (6-carboxyfluorescein)-TTGCAGAACGCCCCTACCCAGAGGC-TAMRA (6-carboxytetramethylrhodamine), on QuantStudio 1 ((Thermo Fisher Scientific, Waltham, MA, USA), as described by Gomes et al. [23]. This study was approved by the ethics committee of the Faculty of Medicine of University of Brasilia (CAAE: 55237816.8.0000.5558).

## 3. Results

**Figure 1:**
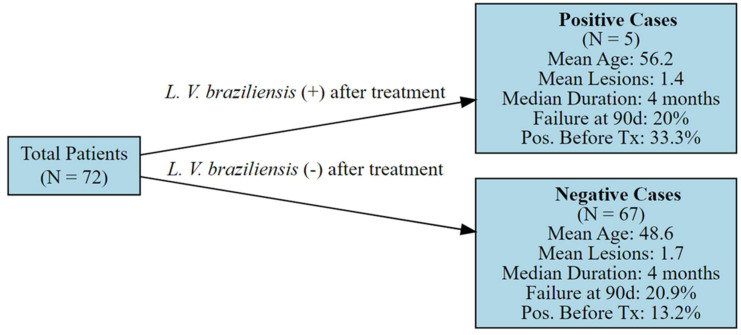
Flow chart showing main characteristics of patients according to the positivity of *L. V. braziliensis* DNA in the nasal mucosa after treatment. n = number of patients. Tx= treatment.

We included 72 patients; of these, *L. V. braziliensis* DNA was found in the nasal mucosa of 5 (7%) patients after treatment and 12 had evidence of mucosal lesions after anterior rhinoscopy (Figure 1). The mean age was 49.2 years and a typical patient had a single lesion with an average four-month duration. In the univariate analysis, only mucosal disease was significantly associated with positivity for *L. V. braziliensis* DNA after treatment. The results are presented in Table 1. The clinical characteristics of patients that presented with mucosal lesions are presented in Table 2.

## 4. Discussion

In this study, we analyzed the presence of *L. V. braziliensis* DNA in the nasal mucosa after treatment. One of the main challenges in the management of a cutaneous lesion caused by this leishmania species is the possible development of mucosal disease at a later stage [24]. Although it is frequently argued that treatment has some protective role in the development of this complication, mucosal disease can still occur in those adequately treated for cutaneous disease [25]. The diagnosis of mucosal lesions is frequently only made a long time after the emergence of a primary cutaneous lesion [26]. Its estimated that over one third of patients will be diagnosed with mucosal disease more than five years after the initial cutaneous ulcer [15]. The development of predictive methods may result in both better management of high-risk patients, including potential modification to the primary treatment and better follow-up, and a reduction in healthcare costs by allowing less frequent visits to low-risk patients and by reducing sequelae resulting from the delayed diagnosis of ML. As leishmaniasis is an infectious disease, it seems to us that the presence of *L. V. braziliensis* in the nasal mucosa is the most sensible potential marker for the development of clinical ML in the future.

We opted to evaluate the presence of the parasite in the nasal mucosa using a kinetoplastic DNA (kDNA) target. Although both DNA and RNA have been used to evaluate the viability of microorganisms [27], in theory, RNA targets are superior in this aspect due to their shorter half-life and lower temperature resistance [28,29]. One potentially important issue is that *L. V. braziliensis* has been shown to present a low proliferative state [30,31], which has been associated with a decline in cellular RNA levels [32] and could therefore limit the sensitivity of this nucleic acid as a target [33]. On the other hand, due to the elevated number of copies of kDNA per parasite, high-sensitivity tests are achieved by targeting this molecule [34]. Importantly, there also seems to be an association of this target and disease activity since a decline in kDNA levels occurs shortly after treatment [35]. Considering these characteristics, we believe that the molecular target used in this study was the most sensible choice to evaluate the presence of leishmania in the nasal mucosa due to its high sensitivity and association with parasite viability.

One of the reasons for the diagnostic time gap between CL and ML is that there is a delay in the development of these lesions, and even patients who did not initially have macroscopic involvement when first evaluated can develop ML at a later stage. The initial oligosymptomatic character of this form also frequently results in a significant gap between the emergence of macroscopic lesion and the diagnosis. This diagnostic delay often results in significant anatomical alterations in nasal and oral anatomy with deformities and chronic symptoms related to the disease [9]. To overcome some of these challenges, research has focused on better stratifying patients based on the potential risk of future development of mucosal lesions so treatment regimen and follow-up can be modified in high-risk patients [36]. One promising strategy is to evaluate the presence of *L. V. braziliensis* genetic material in the nasal mucosa using a swab [20]. In addition to being noninvasive, the collection of samples through swabs has also resulted in highly accurate tests being used in the diagnosis of subclinical involvement of nasal mucosa by leishmania [37,38]. We have, however, to consider that the prognostic value of this test is not really known. One reason is that conducting prognostic studies based on patients with initial cutaneous disease would require long-term follow-up since more than one in twenty mucosal disease cases only develop 15 years after the cutaneous ulcer [15]. Additionally, it is also possible that some patients who will develop mucosal disease may not test positive for *L. V. braziliensis* DNA in the mucosa since the presence of the parasite cannot be shown in all cases due to the limited sensitivity of the PCR test—76% according to a latest metanalysis [39]. Despite these limitations and considering that the presence of leishmania in the mucosa is necessary for the development of ML, we believe that the test used in this study can help to identify patients with a higher risk of developing mucosal disease in the future.

In our study, *L. V. braziliensis* DNA was present in 7% of patients after treatment. The presence of *Leishmania* spp. DNA in the nasal mucosa before treatment has already been shown to be associated with important clinical and immunological characteristics [20,37,40]. After treatment in an *L. V. panamensis* area, Valencia et al. found positivity for *L. V. braziliensis* in the nasal mucosa in more than 63% of the 41 tested CL patients [41]. This high detection rate of the parasite after treatment is likely to result from the initially higher dissemination of this species to the nasal mucosa, this being evidenced by the high positivity rate—88%—before treatment in the study, as compared to rates between 7.8% and 30.3% in *L. V. braziliensis* areas [20,37]. Studies evaluating the presence of the parasite early after treatment in *L. V. braziliensis* areas are lacking, reflecting the under-exploration of this important potential clinical marker in the follow-up and management of ATL patients. Azevedo has shown a 0.9% positivity of leishmania in the nasal mucosa of 330 patients, with collection being performed between the end of treatment and five years after [42]. Similarly to ours, Azevedo’s study was also performed in an *L. V. braziliensis* area, but sample collection was performed a longer time after treatment completion. The lower positivity observed by this author could be associated with a clearance of parasites from the nasal mucosa with time. Its known that parasite load decreases with time [43], leading to lower sensitivity of parasitological tests [44,45,46]. This could mean that the difference in positivity rate observed between this study and ours could be related to the lower sensitivity of the test associated with the longer duration of disease in their study.

We performed a systematic otorhinolaryngological exam of all patients, with or without related complaints. This may explain the lower proportion of patients with grade IV or V mucosal lesions as compared to Lessa’s initial observation, 48% versus 25% in our study [9]. Another explanation could be the difficulty in accessing healthcare in the area of this author’s study resulting in more advanced disease. Importantly, the presence of mucosal disease was significantly associated with the positivity for *L. V. braziliensis* on the nasal swab after treatment with a total of one fourth of patients with mucosal lesions being positive, none of them with clinical signs of active mucosal disease. Although there was a higher mean age of patients with PCR positivity (56.2 years versus 49.2 years in the total population), this difference was of a small magnitude and not statistically significant. This make our findings less likely to be explained by immunosenescence. Contrarily to what occurs in visceral leishmaniasis, where there are concepts of clinical cure and parasitological cure [47], in mucosal and cutaneous leishmaniasis cure is evaluated exclusively with clinical parameters [48,49]. *L. V. braziliensis* antigens have been shown to persist in scars [50] and the parasite can be cultured long after clinical cure in some patients [51]. The association of parasite persistence with a local inflammatory background [50] and the potential relapse of disease after immunosuppression [52] raise doubts about relying only on clinical features to manage mucosal leishmaniasis patients.

One limitation of our study is the association between molecular findings and clinical outcomes. As we did not follow these patients for a long period, we cannot conclude that the presence of *L. V. braziliensis* DNA after treatment can lead to the development or recurrence of mucosal leishmaniasis. Although the presence of *L. V. braziliensis* DNA does not undoubtedly confirm viability, the use of the molecular target we used has already been shown to be associated with disease activity in other Leishmania species causing disease in humans [35].

## 5. Conclusions

We have shown that molecular signatures of the parasite can persist in the nasal mucosa after treatment, and are possibly associated with important clinical characteristics. Whether molecular screening of all patients after treatment should be performed and if positivity for *L. V. braziliensis* in the nasal mucosa at this time point requires modification in treatment or follow-up are questions that need to be answered by further studies.

## Figures and Tables

**Table 1:** Univariate analysis of the association of clinical variables with positivity for *L. V. braziliensis* in the nasal mucosa after treatment.

Variables	Positive for *L. V. braziliensis*	Negative for *L. V. braziliensis*	*p*-Value
Age years (SD) †	56.2(9.6)	48.6(16.2)	0.16
Time since disease onset, median (IQR), months *	4(2)	4(3.5)	0.50 *
Number of lesions (SD) †	1(1)	1(1)	0.84
Sex			
Male (%)	1(20)	41(61.2)	0.15
Female (%)	4(80)	26(38.8)	
Mucosal disease	3(60)	9(13.4)	0.031
Therapeutic failure	1(20)	14(20.9)	1.0
Treatment			
Amphotericin B	1(20)	7(11.1)	0.14
Endovenous Meglumine antimoniate	0	14(22.2)	
Intralesional Meglumine antimoniate	1(20)	28(44.4)	
Miltefosine	3(60)	14(22.2)	

SD = standard deviation; IQR = interquartile range. † Welch two-sample *t*-test; * Wilcoxon rank sum with continuity correction; *p*-values for categorical variables were analyzed using Fisher’s exact test.

**Table 2:** Clinical characteristics of patients with mucosal lesions.

Patient Number	Clinical Findings	Grading ^†^
PCT1	Shallow ulcer with Hematic crust on nasal septum	II
PCT2	Nodules and hyperemia in the nasal septum	I
PCT3	Hyperemia, deep ulcers, septal perforation and nasal architecture alteration	V
PCT4	Hyperemia, hematic crust and shallow ulcers	II
PCT5	Hyperemia, hematic crust and shallow ulcers	II
PCT6	Septal perforation with attached crusts	
PCT7	Wide septal perforation with multiple nodules and nasal tip collapse	V
PCT8	Septal perforation with elevated margins and hematic crusts	IV
PCT9	Bilateral papules at the nasal septum	I
PCT10	Nodules in the right nasal septum	I
PCT11 *	Papules and nodules in gingival and palatal mucosa	I
PCT12	Nodules in nasal septum	I

^†^ according to Lessa’s staging system (I–V); * this patient had lesions only on the oral mucosa and as a consequence classification was applied by analogy.

## Data Availability

The data presented in this study are available on request from the corresponding author.

## References

[1] Alvar J., Vélez I.D., Bern C., Herrero M., Desjeux P., Cano J., Jannin J., den Boer M., Team W.L.C. (2012). Leishmaniasis worldwide and global estimates of its incidence. PLoS ONE.

[2] World Health Organization Leishmaniasis. https://www.who.int/news-room/fact-sheets/detail/leishmaniasis.

[3] Gordón-Núñez M.A., Ferreira S.J., Andrade A.L.D.L.d., Luz K.G., Milan E.P., Galvão H.C. (2014). New World Mucocutaneous Leishmaniasis with Oral Manifestations: Case Report and Damage Repair. Am. J. Infect. Dis..

[4] Costa A.A.U., Saldanha A.C., Leite B.M., Ramos B., Junior I.A., Noronha A.L., Báfica A., Barral A., Corbett C.E., Costa J.M. (2005). Imaging exams of bone lesions in patients with diffuse cutaneous leishmaniasis (DCL). Acta Trop..

[5] Costa J.M.L., Saldanha A.C.R., Silva A.C.d.M., Serra Neto A., Galvão C.E.S., Silva C.d.M.P., Silva A.R.d. (1992). Estado atual da leishmaniose cutânea difusa (LCD) no Estado do Maranhão: II. Aspectos epidemiológicos, clínico-evolutivos. Rev. Da Soc. Bras. De. Med. Trop..

[6] Lessa M.M., Lessa H.A., Castro T.W., Oliveira A., Scherifer A., Machado P.R.L., Carvalho Filho E.M.d. (2007). Leishmaniose mucosa: Aspectos clínicos e epidemiológicos. Rev. Bras. Otorrinolaringol..

[7] Cuentas E.L., Marsden P., Cuba C., Barreto A., Campos M. (1984). Possible risk factors in development of mucosal lesions in leishmaniasis. Lancet.

[8] Machado-Coelho G.L., Caiaffa W.T., Genaro O., Magalhaes P.A., Mayrink W. (2005). Risk factors for mucosal manifestation of American cutaneous leishmaniasis. Trans. R. Soc. Trop. Med. Hyg..

[9] Lessa H.A., Lessa M.M., Guimaraes L.H., Lima C.M., Arruda S., Machado P.R., Carvalho E.M. (2012). A proposed new clinical staging system for patients with mucosal leishmaniasis. Trans. R. Soc. Trop. Med. Hyg..

[10] Turetz M.L., Machado P.R., Ko A.I., Alves F., Bittencourt A., Almeida R.P., Mobashery N., Johnson W.D., Carvalho E.M. (2002). Disseminated leishmaniasis: A new and emerging form of leishmaniasis observed in northeastern Brazil. J. Infect. Dis..

[11] Oliveira M.R.F.d., Macêdo V.d.O., Carvalho E.M.d., Barral A., Marotti J.G., Bittencourt A., Abreu M.V.A.d., Orge Orge M.d.L.G., Lessa H.d.A., Marsden P.D. (1995). Estudo evolutivo da leishmaniose mucosa (7 a 17 anos de seguimento) causada por Leishmania (Viannia) braziliensis em Três Braços, Bahia. Rev. Soc. Bras. Med. Trop..

[12] da Costa D.C.S., Palmeiro M.R., Moreira J.S., da Costa Martins A.C., da Silva A.F., de Fátima Madeira M., Quintella L.P., Confort E.M., de Oliveira Schubach A., da Conceição Silva F. (2014). Oral manifestations in the American tegumentary leishmaniasis. PLoS ONE.

[13] Borges K.T., Nogueira L.S.C., Sampaio J.H.D., Tauil P.L., Sampaio R.N.R. (2005). Clinical, epidemiological and therapeuthic study of 402 patients with american cutaneous leishmaniasis attended at University Hospital of Brasilia, DF, Brazil. Bras. Dermatol..

[14] García Choque M.A. (2015). Leishmaniasis con afectación de vía aérea inferior y superior, sin compromiso cutáneo. Rev. Am. Med. Respir..

[15] Marsden P.D., Llanos-Cuentas E.A., Lago E.L., Cuba C.C., Barreto A.C., Costa J.M., Jones T.C. (1984). Human mucocutaneous leishmaniasis in Três Braços, Bahia-Brazil. An area of *Leishmania braziliensis braziliensis* transmission. III-Mucosal disease presentation and initial evolution. Rev. Soc. Bras. Med. Trop..

[16] Ruas A.C.N., Lucena M.M., da Costa A.D., Vieira J.R., de Araújo-Melo M.H., Terceiro B.R.F., de Sousa Torraca T.S., de Oliveira Schubach A., Valete-Rosalino C.M. (2014). Voice disorders in mucosal leishmaniasis. PLoS ONE.

[17] Mota L.A.A., Miranda R.R. (2011). Manifestações dermatológicas e otorrinolaringológicas na Leishmaniose. Arq. Int. Otorrinolaryngol..

[18] Silveira F.T. (2019). What makes mucosal and anergic diffuse cutaneous leishmaniases so clinically and immunopathogically different? A review in Brazil. Trans. R. Soc. Trop. Med. Hyg..

[19] Meneses A.M.d. (2007). Perfil Epidemiológico, Clínico e Terapêutico dos Pacientes com a Forma Mucosa de Leishmaniose Tegumentar Americana, Atendidos no Instituto de Pesquisa Clínica Evandro Chagas-Fundação Oswaldo Cruz, Rio de Janeiro, no Período de 1989 a 2004. Ph.D. Thesis.

[20] Barroso D.H., Nóbrega O.d.T., de Araújo C.N., Freire G.S.M., Martins S.S., Rodrigues B.C., Gomes C.M., Sampaio R.N.R. (2021). The Presence of Leishmania braziliensis DNA in the Nasal Mucosa of Cutaneous Leishmaniasis Patients and the Search for Possible Clinical and Immunological Patterns of Disease Progression: A Cross Sectional Study. Front. Cell. Infect. Microbiol..

[21] Gomes C.M. (2014). Acurácia da Reação em Cadeia da Polimerase em Amostras de Saliva, Swab Nasal e Papel Filtro Oral no Diagnóstico da Leishmaniose Tegumentar Americana: Estudo Clínico, Revisão Sistemática da Literatura e Meta-Análise.

[22] Ministério da Saúde, Secretaria de Vigilancia em Saúde, Departamento de Vigilância Epidemiológica (2017). Manual de Vigilância e Controle da Leishmaniose Tegumentar Americana Atualizada.

[23] Gomes C.M., Cesetti M.V., de Paula N.A., Vernal S., Gupta G., Sampaio R.N., Roselino A.M. (2017). Field Validation of SYBR Green- and TaqMan-Based Real-Time PCR Using Biopsy and Swab Samples To Diagnose American Tegumentary Leishmaniasis in an Area Where Leishmania (Viannia) braziliensis Is Endemic. J. Clin. Microbiol..

[24] Amato V.S., Tuon F.F., Bacha H.A., Neto V.A., Nicodemo A.C. (2008). Mucosal leishmaniasis: Current scenario and prospects for treatment. Acta Trop..

[25] Oliveira C.C., Lacerda H.G., Martins D.R., Barbosa J.D., Monteiro G.R., Queiroz J.W., Sousa J.M., Ximenes M.F., Jeronimo S.M. (2004). Changing epidemiology of American cutaneous leishmaniasis (ACL) in Brazil: A disease of the urban–rural interface. Acta Trop..

[26] Schleucher R.D., Zanger P., Gaessler M., Knobloch J. (2008). Successful diagnosis and treatment 50 years after exposure: Is mucocutaneous leishmaniasis still a neglected differential diagnosis?. J. Travel Med..

[27] Keer J., Birch L. (2003). Molecular methods for the assessment of bacterial viability. J. Microbiol. Methods.

[28] Sheridan G., Szabo E., Mackey B. (1999). Effect of post-treatment holding conditions on detection of tufA mRNA in ethanol-treated Escherichia coli: Implications for RT-PCR-based indirect viability tests. Lett. Appl. Microbiol..

[29] Mohapatra S. (2014). Drug resistance in leishmaniasis: Newer developments. Trop. Parasitol..

[30] Mandell M.A., Beverley S.M. (2017). Continual renewal and replication of persistent *Leishmania major* parasites in concomitantly immune hosts. Proc. Natl. Acad. Sci. USA.

[31] Saunders E.C., Ng W.W., Kloehn J., Chambers J.M., Ng M., McConville M.J. (2014). Induction of a stringent metabolic response in intracellular stages of Leishmania mexicana leads to increased dependence on mitochondrial metabolism. PLoS Pathog..

[32] Jara M., Berg M., Caljon G., de Muylder G., Cuypers B., Castillo D., Maes I., Orozco M.d.C., Vanaerschot M., Dujardin J.-C. (2017). Macromolecular biosynthetic parameters and metabolic profile in different life stages of Leishmania braziliensis: Amastigotes as a functionally less active stage. PLoS ONE.

[33] González-Escalona N., Fey A., Höfle M.G., Espejo R.T., Guzmán C.A. (2006). Quantitative reverse transcription polymerase chain reaction analysis of Vibrio cholerae cells entering the viable but non-culturable state and starvation in response to cold shock. Environ. Microbiol..

[34] Mary C., Faraut F., Lascombe L., Dumon H. (2004). Quantification of Leishmania infantum DNA by a real-time PCR assay with high sensitivity. J. Clin. Microbiol..

[35] Disch J., Oliveira M.C., Orsini M., Rabello A. (2004). Rapid clearance of circulating Leishmania kinetoplast DNA after treatment of visceral leishmaniasis. Acta Trop..

[36] Guerreiro J.B., Cruz Á.A., Barral A., Lessa H.A., Rocha H., Carvalho E.M. (2000). Mucosal leishmaniasis: Quantitative nasal cytology as a marker of disease activity and indicator of healing. Ann. Otol. Rhinol. Laryngol..

[37] Canário A., Queiroz M., Cunha G., Cavalcante T., Riesz V., Sharma R., De Noronha A., Correia T., Barral-Netto M., Barral A. (2019). Presence of parasite DNA in clinically unaffected nasal mucosa during cutaneous leishmaniasis caused by Leishmania (Viannia) braziliensis. Clin. Microbiol. Infect..

[38] Adams E.R., Gomez M.A., Scheske L., Rios R., Marquez R., Cossio A., Albertini A., Schallig H., Saravia N.G. (2014). Sensitive diagnosis of cutaneous leishmaniasis by lesion swab sampling coupled to qPCR. Parasitology.

[39] Gomes C.M., Mazin S.C., Santos E.R.d., Cesetti M.V., Bächtold G.A.B., Cordeiro J.H.d.F., Theodoro F.C.E.T., Damasco F.d.S., Carranza S.A.V., Santos A.d.O. (2015). Accuracy of mucocutaneous leishmaniasis diagnosis using polymerase chain reaction: Systematic literature review and meta-analysis. Memórias Do Inst. Oswaldo Cruz.

[40] Figueroa R.A., Lozano L.E., Romero I.C., Cardona M.T., Prager M., Pacheco R., Diaz Y.R., Tellez J.A., Saravia N.G. (2009). Detection of Leishmania in unaffected mucosal tissues of patients with cutaneous leishmaniasis caused by Leishmania (Viannia) species. J. Infect. Dis..

[41] Martínez-Valencia A.J., Daza-Rivera C.F., Rosales-Chilama M., Cossio A., Rincón E.J.C., Desai M.M., Saravia N.G., Gómez M.A. (2017). Clinical and parasitological factors in parasite persistence after treatment and clinical cure of cutaneous leishmaniasis. PLoS Neglected Trop. Dis..

[42] Azevedo A.C.A. (2019). Análise do Tropismo de Leishmania Braziliensis Para a Mucosa Nasal Antes e Longo Período Após o Tratamento da Leishmaniose Cutânea Localizada. Doctoral Thesis.

[43] Pereira L.d.O.R., Barbosa R. (2017). Is Leishmania (Viannia) braziliensis parasite load. World Health.

[44] Hosseinzadeh M., Omidifar N., Lohrasb M.H. (2012). Use of fine needle aspiration cytology in the diagnosis of cutaneous leishmaniasis: A comparison with the conventional scraping method. Trop. Dr..

[45] Reis L.d.C., Brito M.E.F.d., Almeida É.L.d., Félix S.M., Medeiros Â.C.R., Silva C.J., Pereira V.R.A. (2008). Clinical, epidemiological and laboratory aspects of patients with American cutaneous leishmaniasis in the State of Pernambuco. Rev. Da Soc. Bras. De. Med. Trop..

[46] Andresen K., Gaafar A., El-Hassan A., Ismail A., Dafalla M., Theander T., Kharazmi A. (1996). Evaluation of the polymerase chain reaction in the diagnosis of cutaneous leishmaniasis due to Leishmania major: A comparison with direct microscopy of smears and sections from lesions. Trans. R. Soc. Trop. Med. Hyg..

[47] Cota G.F., de Sousa M.R., Fereguetti T.O., Rabello A. (2013). Efficacy of anti-leishmania therapy in visceral leishmaniasis among HIV infected patients: A systematic review with indirect comparison. PLoS Neglected Trop. Dis..

[48] Carvalho J.d.P., Silva S.N., Freire M.L., Alves L.L., Souza C.S.A.d., Cota G. (2022). The cure rate after different treatments for mucosal leishmaniasis in the Americas: A systematic review. PLOS Neglected Trop. Dis..

[49] Olliaro P., Grogl M., Boni M., Carvalho E.M., Chebli H., Cisse M., Diro E., Cota G.F., Erber A.C., Gadisa E. (2018). Harmonized clinical trial methodologies for localized cutaneous leishmaniasis and potential for extensive network with capacities for clinical evaluation. PLoS Neglected Trop. Dis..

[50] Amato V.S., Andrade Jr H.F., Neto V.A., Duarte M.I.S. (2003). Persistence of tumor necrosis factor-α in situ after lesion healing in mucosal leishmaniasis. Am. J. Trop. Med. Hyg..

[51] Schubach A., Marzochi M., Cuzzi-Maya T., Oliveira A.V., Araujo M.L., Oliveira A., Pacheco R.S., Momen H., Conceicao-Silva F., Coutinho S.G. (1998). Cutaneous scars in American tegumentary leishmaniasis patients: A site of Leishmania (Viannia) braziliensis persistence and viability eleven years after antimonial therapy and clinical cure. Am. J. Trop. Med. Hyg..

[52] Santos G.d.A., Sousa J.M., Aguiar A.H.B.M.d., Torres K.C.S., Coelho A.J.S., Ferreira A.L., Lima M.I.S. (2023). Systematic Review of Treatment Failure and Clinical Relapses in Leishmaniasis from a Multifactorial Perspective: Clinical Aspects, Factors Associated with the Parasite and Host. Trop. Med. Infect. Dis..

